# HMGA2 promotes resistance against paclitaxel by targeting the p53 signaling pathway in colorectal cancer cells

**DOI:** 10.1016/j.heliyon.2024.e31431

**Published:** 2024-05-20

**Authors:** Haizhong Jiang, Xueying Li, Feng Zhou, Yang Xi, Guoqiang Xu

**Affiliations:** a Department of Gastroenterology, First Affiliated Hospital, School of Medicine, Zhejiang University, 310003, Hangzhou, China; b Department of Gastroenterology, First Affiliated Hospital, Ningbo University, Ningbo, Zhejiang, 315000, China; c Institute of Biochemistry and Molecular Biology, School of Medicine, Ningbo University, Ningbo, Zhejiang, 315211, China

**Keywords:** HMGA2, Colorectal cancer, Paclitaxel, p53 signaling

## Abstract

Colorectal cancer is one of the most common malignancies and ranks second in terms of cancer-related mortality worldwide due to its metastasis, drug resistance, and reoccurrence. High-mobility gene group A2 (HMGA2) is overexpressed in colorectal cancer, contributing to the aggressiveness of tumor malignance, and promotes drug resistance in many types of cancer. However, the underlying molecular mechanism of HMGA2 is yet to be elucidated. In this study, we showed that HMGA2 is overexpressed in colorectal cancer tissue, and knockdown of HMGA2 significantly inhibited colorectal cancer cell growth and migratory capability. HMGA2 regulates the cancer cell response to a widely used anti-cancer drug, paclitaxel (PTX). HMGA2 knockdown increased cell death, whereas HMGA2 overexpression decreased cell death after PTX treatment. Furthermore, lower reactive oxygen species (ROS) levels and mitochondrial potential were detected in HMGA2 overexpression cells after PTX treatment. However, HMGA2 knockdown produced the opposite effect. RNA sequencing showed a p53 signaling pathway-dependent regulation in HMGA2 knockdown cells. Combined with p53 inhibitors and HMGA2 knockdown, a synergetic effect of more cell death was observed in colorectal cancer cells after PTX treatment. Thus, we showed that HMGA2 can activate p53 signaling to regulate colorectal cancer cell death after PTX treatment. Altogether, our results reveal novel insights into the molecular mechanisms underlying HMGA2-mediated cancer cell resistance against PTX and highlight the potential of targeting HMGA2 and p53 signaling for the therapeutic investigation of colorectal cancer.

## 1 Introduction

As one of the most common malignancies worldwide, colorectal cancer is a serious threat to human health. The global cancer statistics (2020) reported that colorectal ranks third in terms of cancer incidence (10.0 %) and second in terms of cancer-related mortalities worldwide (9.4 %) [1]. The numbers of new cases and deaths were approximately 1148515 (6 %) and 576858 (5.8 %), respectively, in 2020 worldwide [2]. A 73.4 % increase in colorectal cancer-related deaths is estimated, which will increase from 0.9 million deaths in 2020 to over 1.6 million deaths by 2040 [3]. Although colorectal cancer mortality has decreased due to early detection and treatment, the five-year survival rate is low, especially in patients with advanced stages of cancer [4]. Compared with the 90 % survival at an early stage, the five-year survival of patients at later stages is only approximately 10 %. Therefore, it is urgent and important to uncover the molecular mechanism of colorectal cancer progression and therapy target.

High-mobility group AT-hook 2A (HMGA2) protein is a member of the high-mobility group (HMG) family, which contains three AT-hooks that can bind to the AT-rich sequence in the DNA minor groove and functions as an architectural transcriptional factor [5,6]. HMGA2 is either lowly expressed or not expressed in adult tissues, whereas it is highly expressed in malignant or cancer cells, implying the oncogenic role of HMGA2. HMGA2 overexpression has been found in most types of human cancers [[7], [8], [9]], including lung, colorectal, ovarian, and gastric cancers. Furthermore, HMGA2 overexpression is associated with poor prognosis, high tumor grade, and lymph node metastasis. As an architectural factor, HMGA2 can alter the chromosome conformation by changing the assembly of the nuclear macromolecular complex via protein–protein or protein–DNA interaction to improve or inhibit transcription of downstream targets [10]. HMGA2 exhibits its oncogenicity in several ways, such as by activating the cell cycle [11,12], promoting epithelial-to-mesenchymal transition (EMT) and metastasis [13,14], and inhibiting apoptosis [15].

Reactive oxygen species (ROS) is an intrinsic byproduct of cell metabolism, which is mainly produced in the mitochondria [16]. Some amount of ROS can benefit cell proliferation and differentiation [17]. However, high ROS levels can induce oxidative stress to trigger cell death. Thus, the induction of ROS overload is an important anti-cancer therapy strategy for cancer [18]. However, most cancer cells can escape chemical drug treatment and develop resistance against oxidative stress, leading to escape from chemotherapy [18,19]. Therefore, targeting the key regulatory factors and demonstrate the molecular mechanism would greatly benefit the chemotherapy of cancer.

In this study, we found that HMGA2 was overexpressed in colorectal cancer tissues. HMGA2 overexpression decreased the ROS levels in PTX-treated colorectal cancer cells via the p53 signaling pathway. Our results provide a basic molecular mechanism of HMGA2 and shed light on drug application for colorectal cancer therapy.

## 2 Material and methods

### 2.1 Patient samples

Fresh tissues of clinical colon cancer were used in this study. Informed consent was obtained from all participating patients, and the study was approved by The Research Ethics Committee (2021-R167) of the First Affiliated Hospital of Ningbo University. The clinical characteristics of colorectal cancer patients are listed in Table 1.

**Table 1:** Clinicopathological characteristics of CRC patients.

	CRC CRC tissue（n = 20） Normal tissue（n = 20）	
Age, mean ± SD,years	66.2 ± 9.22	
Sex	Male (65 %)	Female(35 %)
Location	Rectum(75 %)	Colon(25 %)
Lymphatic invasion	10/20（50 %）	
Venous invasion	12/20(60 %)	
Perineural invasion	4/20(20 %)	

CRC, colorectal cancer.

### 2.2 Cell culture and treatments

Routinely used human colorectal cancer cell lines, including HCT116, RKO, and SW620, were cultured in RPMI 1640 medium containing 10 % fetal bovine serum (Sigma-Aldrich, Shanghai, China) in the incubator with 5 % CO_2_ at 37 °C. PTX (HY–B0015), 5-fluorouracil (5-FU, HY-90006), cisplatin (HY-17394), capecitabine (HY–B0016), Pifithrin-μ (HY-10940), and Pifithrin-β (HY-16702) were purchased from MedChemExpress (Shanghai, China). Dimethyl sulfoxide (DMSO) was used as the solution for reagents. For cell treatments, the treated time and concentration were indicated in the results and DMSO was used as the control.

### 2.3 Lentiviral infection, siRNAs, and transfection

To overexpress HMGA2, a lentivirus carrying the HMGA2 CDS (protein-coding region) was purchased from GeneChem Biotechnology (Shanghai, China). The blank vector was used as the control MOCK. The following siRNAs were used for HMGA2: siA2–1: 5′-CGGCCAAGAGGCAGACCUATT-3′, siA2–2: 5′-CCGGUGAGCCCUCUCCUAATT-3′, and siA2–3: 5′-GUCCCUCUAAAGCAGCUCATT-3′ [20]. The none-specific siRNA was used as the control siCtrl. For the siRNA transfection, Lipofectamine® RNAiMAX Transfection Reagent (Invitrogen, Germany) was used according to the manufacturer's protocol.

### 2.4 RNA sequencing (RNA-seq) analysis

For RNA-seq, total RNAs were extracted using TRIzol reagent (Invitrogen, Carlsbad, CA, USA) following the manufacturer's protocol and verified by Bioanalyzer 2100 (Agilent, CA, USA) to fulfill the standard for sequencing by LC Bio-Technology (Hangzhou, China). Triplicate samples were established independently for all assays of the sequencing data. HISAT2 (https://ccb.jhu.edu/software/hisat2) was used to map reads to the reference genome of *Homo sapiens* GRCh38. We used StringTie to evaluate the expression of mRNAs by calculating FPKM (FPKM = [total_exon_fragments/mapped_reads(millions) × exon_length(kB)]). The differentially expressed mRNAs were selected with fold change >2 or fold change <0.5 and with parametric F-test comparing nested linear models (p-value <0.05) by R package edgeR (https://bioconductor.org/packages/release/bioc/htmL/edgeR.html). The pathway enrichment analysis was evaluated based on the Kyoto Encyclopedia of Genes and Genomes database (KEGG). RNA-seq data were uploaded to the National Genomics Data Center of China (NGDC) with the accession number #PRJCA020847.

### 2.5 Western blotting

Total proteins were extracted using a standard RIPA lysis buffer containing proteinase cocktail (Solarbio, China). After separating with 10 % sodium dodecyl sulfate–polyacrylamide gel electrophoresis gel, proteins were transferred to the PVDF membrane. The primary antibodies were p53 (AB76242, Abcam), HMGA2 (A24392, ABclonal), and β-actin (AC026, ABclonal).

### 2.6 Measurement of ROS and mitochondrial integrity

Cells were incubated with 2′,7′-Dichlorodihydrofluorescein diacetate (DCFH-DA, HY-D0940, MCE, China) for 30 min at 37 °C, then cellular ROS levels were assessed using a flow cytometer. For the detection of mitochondrial integrity, the mitochondrial membrane potential (Δψ) was measured using the fluorescent dye JC-1 (BD, USA) according to the manufacturer's protocol. The samples were analyzed using a flow cytometer.

### 2.7 Cell proliferation and migratory capability assays

Cell proliferation was measured using a Cell Counting Kit-8 (HY–K0301, MCE, China) according to the manufacturer's protocol. The cells were plated in at least three independent wells of 96-well plates, and the absorbance was measured at 490 nm. The migratory capability of the cells was analyzed by performing a Transwell experiment and visualized using a microscope (magnification, × 10) at 72 h.

### 2.8 Statistical analysis

Three independent replicates were performed, and the results are presented as the mean ± standard deviation (SD). Statistical tests were analyzed using Student's *t*-test or two-way ANOVA for group analysis in GraphPad Prism 8.3 software, and a p-value <0.05 was considered statistically significant.

## 3 Results

### 3.1 HMGA2 is overexpressed in colorectal cancer and promotes cancer cell growth and migratory capability

**Fig. 1:**
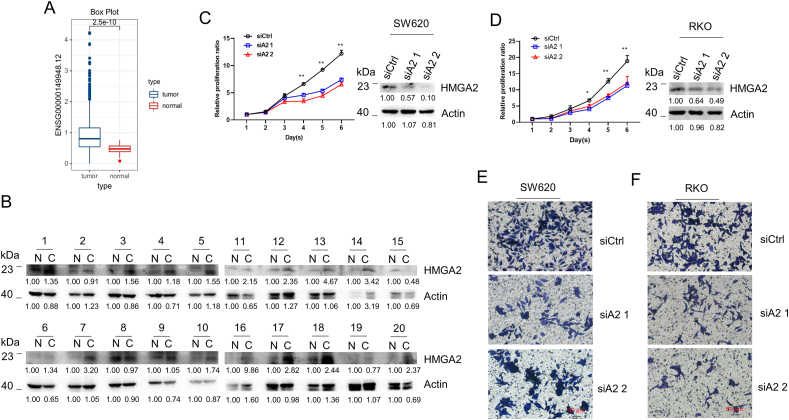
HMGA2 is overexpressed in colorectal cancer and promotes cancer cell growth and metastasis **A**. The transcriptional level of HMGA2 between tumor and normal tissue in colon adenocarcinoma (COAD) from The Cancer Genome Atlas Program (TCGA) database. **B**. HMGA2 protein expression between tumor and para-non tumor samples based on Western blotting analysis. N represents normal. C represents cancer; The cell growth of SW620 (**C**) and RKO cells (**D**) with siRNA mediated HMGA2 knockdown based on CCK-8 analysis. Two pairs of siRNAs as siA2 1 and siA2 2 were applied for HMGA2 knockdown. The none-specific siRNA was used as the control siCtrl. The corresponding knockdown efficiency was identified by Western blot. The density of immunoblot was analyzed by Image J soft. The metastasis of SW620 (**E**) and RKO cells (**F**) with HMGA2 knockdown based on Transwell experiments. Each analysis was presented after three individual repeats. *P < 0.05; * *P < 0.01.

We investigated and compared the expression of HMGA2 between tumor and normal tissue in colon adenocarcinoma (COAD) from The Cancer Genome Atlas Program (TCGA), and a higher HMGA2 expression (p = 2.5e−10) was found (Fig. 1A). Furthermore, a distinctly higher level of HMGA2 protein was also found in clinical tumor samples (Fig. 1B). To investigate the role of HMGA2, we synthesized HMGA2 siRNAs and downregulated the expression of HMGA2 in human colorectal cells (Fig. 1C and D). Compared with the control, SW620 (Fig. 1C) and RKO (Fig. 1D) with HMGA2 knockdown showed decreased cell growth. Furthermore, fewer migratory cells were observed in HMGA2 knockdown cells compared with that in the control cells (Fig. 1E&F).

### 3.2 HMGA2 alleviates PTX treatment in colorectal cancer cells

**Fig. 2:**
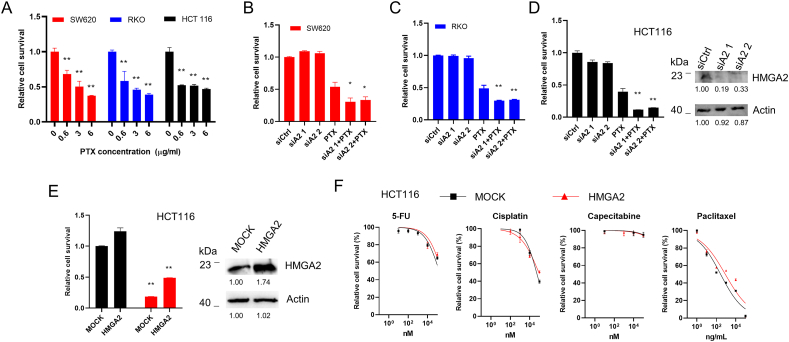
HMGA2 alleviates paclitaxel treatment **A**. The cell survival analysis of three colorectal cancer cell lines of SW620, RKO, and HCT116 treated with different concentrations of paclitaxel (PTX) for 24 h; The cell survival analysis of SW620 cells indicated by red color (**B**), RKO cells indicated by blue color (**C**), and HCT116 cells indicated by black color (**D**) with 2 μg/ml PTX treatment after siRNA mediated HMGA2 knockdown. Two pairs of siRNAs as siA2 1 and siA2 2 were applied for HMGA2 knockdown. The none-specific siRNA was used as the control siCtrl. The corresponding knockdown efficiency in HCT116 cells was identified by Western blot. **E**. The cell survival analysis of HCT116 cells with PTX treatment after HMGA2 overexpression. The HMGA2 overexpression was identified by Western blot. **F.** The toxicity of clinical drugs 5-FU, cisplatin, capecitabine, and PTX on HCT116 cells with HMGA2 overexpression was evaluated after 24 h treatment. Each analysis was presented after three individual repeats. *P < 0.05; * *P < 0.01.

PTX is a widely used clinical drug. However, drug resistance is often found in patients with colorectal cancer. To determine whether HMGA2 affects cell sensitivity toward PTX, we treated colorectal cancer cells with PTX. As shown in Fig. 2A, PTX could kill cancer cells in a concentration-dependent way. Furthermore, we found that HMGA2 knockdown promoted the PTX effects on cell death in three different tested cell lines, namely SW620 (Fig. 2B), RKO (Fig. 2C), and HCT116 cells (Fig. 2D). However, HMGA2 overexpression significantly alleviated the PTX effects (Fig. 2E). The efficiency of HMGA2 siRNA knockdown and overexpression were identified and shown by performing western blotting (Fig. 2D&E). These results suggest that HMGA2 alleviates PTX treatment and can be targeted in cancer therapy. We further detected the influence of HMGA2 overexpression on several clinical drugs. As displaced in Fig. 2F, overexpression of HMGA2 showed no effects of capecitabine, increased the sensitivity in low concentration, while decreased the sensitivity in high concentration of cisplatin. HMGA2 showed the increased resistant to 5-FU and PTX in a various concentration. However, there had limited influence only when 5-FU was applied with high concentration of 500 μM which still had low cellular toxicity to cells. Thus, we further focused on PTX treatment.

### 3.3 HMGA2 decreases cellular oxidative stress after PTX treatment

**Fig. 3:**
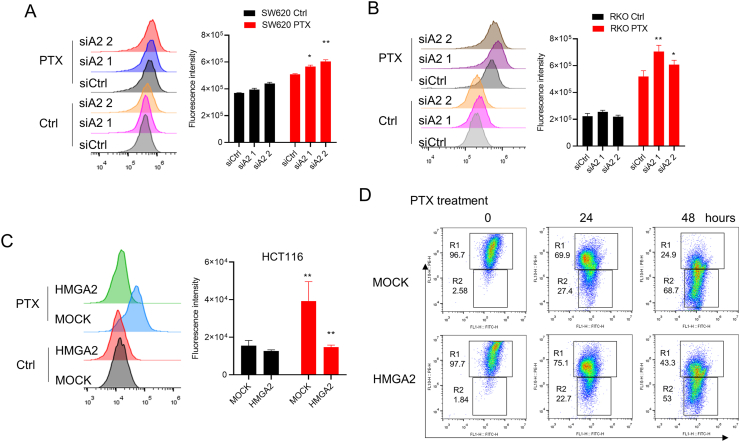
HMGA2 decreases cellular oxidative stress after paclitaxel (PTX) treatment Cellular ROS detection of SW620 cells (**A**) and RKO cells (**B**) after siRNA mediated HMGA2 knockdown with or without 2 μg/ml PTX for 24 h treatment based on 2′,7′-Dichlorodihydrofluorescein diacetate (DCFH-DA) staining and flow cytometry analysis. **C**. Cellular ROS detection of HCT116 cells after HMGA2 overexpression with or without PTX treatment for 24 h based on DCFH-DA staining and flow cytometry analysis. **D**. The mitochondrial potential detection indicated by JC-1 staining in the HMGA2 overexpressed HCT166 cells with or without PTX treatment for 24 and 48 h. Each analysis was presented after three individual repeats. *P < 0.05; * *P < 0.01.

PTX can directly induce cellular ROS accumulation. We found significantly increased ROS levels after PTX treatment in three different cancer cell lines, namely SW620 (Fig. 3A), RKO cells (Fig. 3B), and HCT116 (Fig. 3C). However, we found more significantly increased ROS in HMGA2 knockdown cells than in the control siRNA-treated cells after PTX treatment in SW620 (Fig. 3A) and RKO (Fig. 3B). However, decreased ROS was found in HMGA2 overexpressed cells than in the control MOCK cells after PTX treatment (Fig. 3C). Moreover, obvious higher or lower ROS levels were found in HMGA2 knockdown or overexpressed cells compared with those in the control cells in normal growth condition, respectively. Furthermore, a decreased mitochondrial potential was observed, as shown by the percent of JC-1 staining (FITC positive only) in the HMGA2 overexpressed cells (11.5 %) compared with that in the MOCK cells (15.1 %) after PTX treatment (Fig. 3D). These results indicate that HMGA2 can decrease cellular ROS level and indicate the importance of HMGA2 in ROS regulation.

### 3.4 HMGA2 alternates p53 signaling

**Fig. 4:**
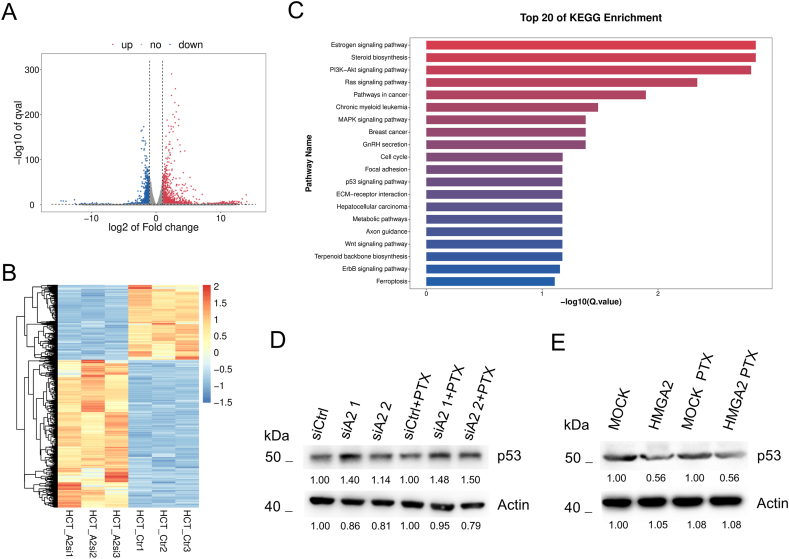
HMGA2 alternates p53 signaling Volcano map (**A**) and heatmap (**B**) of RNA sequence results after siRNA mediated HMGA2 knockdown in HCT116 cells. **C**. The KEGG analysis of obviously differential expressed genes was collected from the comparison between HMGA2 knockdown and corresponding siCtrl cells. Identification of p53 expression in HMGA2 knockdown (**D**) or overexpression cells (**E**) with or without PTX treatment based on Western blot.

To give a further overview of the cellular effects induced by HMGA2, we performed an mRNA sequencing analysis. In total, 2929 genes were significantly changed, with 1944 upregulated and 985 downregulated (Fig. 4A). A fine correlation among samples was shown using the heatmap of changed genes (Fig. 4B). The significantly changed expression genes were collected and analyzed using KEGG analysis. Among the top twenty of KEGG enrichment, the estrogen signaling pathway was the first one to be correlated with HMGA2 knockdown. This was also supported by a recent reference that HMGA2 is a key oncogene in breast cancer [21], indicating the reliability of our results. We found that the phosphoinositide-3-kinase (PI3K)-Akt signaling pathway was the third significant enriched pathway, and the p53 signaling pathway was also enriched. Notably, PI3K-AKT signaling is an environmental response, and p53 is an important downstream conductor [22]. Therefore, we investigated whether HMGA2 alteration can regulate p53 expression. As shown in Fig. 4D, HMGA2 knockdown significantly increased the p53 protein levels. However, its levels were decreased in HMGA2 overexpression cells (Fig. 4E).

### 3.5 Inhibition of p53 has synergistic effects on cell survival in HMGA2 knockdown cells

**Fig. 5:**
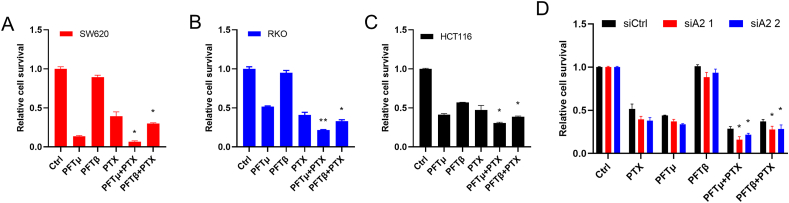
Inhibition of p53 exhibits synergistic effects on cell survival in HMGA2 knockdown cells The cell survival analysis of SW620 cells indicated by red color (**A**), RKO cells indicated by blue color (**B**), and HCT116 cells indicated by black color (**C**) treated with p53 inhibitors PFTμ and PFTβ, and PTX for 24 h. **D**. The cell survival analysis of RKO cells treated with HMGA2 knockdown and/or p53 inhibitors PFTμ and PFTβ with PTX for 24 h. Black color represents siCtrl, red and blue colors represent HMGA2 knockdown of siA2 1 and siA2 2, respectively. Each analysis was presented after three individual repeats. *P < 0.05; * *P < 0.01.

To further confirm the significance of p53 in HMGA2 regulation, cells were treated with widely used p53 inhibitors PFTμ and PFTβ. Both inhibitors showed inhibition on cell growth, although PFTμ exhibited a stronger inhibition than PFTβ (Fig. 5A–C). Furthermore, a significantly decreased cell survival was observed in the combination of PTX with p53 inhibitors in tested three cell lines, SW620 (Fig. 5A), RKO (Fig. 5B), and HCT116 cells (Fig. 5C). Furthermore, the combination of HMGA2 knockdown and p53 inhibitors, PFTμ or PFTβ, exhibited distinct effects on cell death after PTX treatment compared with the corresponding control treatments (Fig. 5D).

## 4 Discussion

As the second rank of cancer-related deaths, more than 935,000 colorectal cancer deaths occurred in 2020 worldwide. The colorectal cancer burden is predicted to reach 3.2 million new cases and 1.6 million deaths by 2040 [2,3]. Moreover, the survival of patients with colorectal cancer is strongly dependent on the cancer stage at diagnosis. The 5-year survival rate for the localized stage is approximately 90 %, whereas that of the distant stage is less than 10 % [23]. Among the people diagnosed with colorectal cancer, 20 % have metastatic cancer, and 40 % present with recurrence after previously treated localized disease [24]. Therefore, there is an urgent need to elucidate the molecular mechanisms underlying the progression of colorectal cancer [4].

During cancer development, cellular ROS plays a crucial role. The intracellular level of ROS is critical for the regulation of cell proliferation and death [16]. ROS can accelerate proliferation, survival, and metastasis by inducing DNA mutations and genomic instability or as a signal molecule for regulatory pathways [18]. However, overloaded ROS can promote oxidative stress, resulting in oxidative damage and cell death [25]. Thus, chemical drugs that induce excessive ROS were considered to be an important therapeutic strategy for cancer treatment. PTX is an antineoplastic agent and is a clinically widely used chemotherapeutic agent for the treatment of advanced solid carcinomas, such as breast, lung, and ovary cancer [26]. In this study, we detected the increased ROS level after PTX treatment and altered mitochondrial membrane potential in colorectal cancer cells, suggesting the common molecular mechanism in different cancer cells of PTX. Our results are consistent with the results of a previous study that showed that PTX-induced ROS accumulation stimulates the expression of HIF-1α and activates caspase-3 signaling to promote apoptosis in prostate cancer cells [27]. Notably, PTX can bind to β-tubulin and prevent the dissociation of microtubules, blocking cell cycle progression, and also inducing apoptosis and senescence through the distinct signaling pathways involved in different cancer cells [28,29].

HMGA2 overexpression has been and is associated with poor prognosis, low survival rates, and advanced stage in various types of human cancer. HMGA2 protein is an independent indicator of shorter patient survival time and tumor progression in several cancers, including gastric cancer, oral squamous cell carcinoma, and bladder cancer [28]. The mouse that had the intestine-specific Hmga2 overexpression accelerated azoxymethane (AOM)/dextran sodium sulfate (DSS) carcinogen-induced tumor development [30], suggesting an important role of HMGA2 in colorectal cancer development. In this study, we found a higher expression of HMGA2 in colorectal cancer tissue than in the *para*-nontumor parts, and decreased cell growth was also confirmed. Our results further highlight the importance of HMGA2 in colorectal cancer.

HMGA2 is an architectural nuclear factor involved in gene transcription and chromatin remodeling. The HMGA2 protein can exert oncogenicity in several ways, such as activation of the cell cycle, enhancement of transcriptional factor activity, maintenance of self-renewing activity of stem cells, interference with apoptosis, and impairment of DNA repair, which is dependent on the downstream genes. HMGA2 can inhibit the expression of p16INK4A and p19ARF to inhibit cellular senescence and maintain stem cell properties [31,32]. Yu et al. showed that HMGA2 improved proliferation by inducing the pI3K/AKT/mTOR/p70S6K signaling and inhibiting p21CIP1/WAF1 expression [32]. In this study, we found that HMGA2 plays a role in regulating the resistance of PTX treatment, except for the cell growth regulation. HMGA2 overexpression alleviates the PTX effects on cell death, whereas HMGA2 knockdown increases the sensitivity of PTX treatment. Mechanically, we found that HMGA2 protein expression was negatively correlated with p53 protein expression. HMGA2 overexpression activated the p53 signaling, as shown by the RNA sequencing analysis. HMGA2 can physically interact with MDM2 and p53. HMGA2 decreased the protein stability of p53 in an MDM2-mediated p53 ubiquitination manner [31,33], which showed a good association with our data and further suggests the reliability of our results and the importance of HMGA2 in p53 regulation.

Previous studies have suggested that HMGA2 overexpression can play critical roles in chemoresistance in various cancers, such as promoting 5-FU drug resistance in gastric and colorectal cancers [34,35]. As an indicated important regulator for drug resistance, the role of HMGA2 in oxidative regulation is largely unknown. In this study, we showed that HMGA2 overexpressing cells showed decreased cellular ROS levels by modulating the mitochondrial membrane potential, which affects the cellular ROS level. However, we did not detect a mitochondrial localization and HMGA2 expression (data not shown), excluding the possibility of a direct influence on mitochondria function. Localization of HMGA2 was mainly restricted to the nucleus; thus, there was an indirect regulation.

p53 is one of the most famous tumor suppressors and protects the cells to prevent tumorigenesis in multiple mechanisms [36]. p53 can promote apoptosis via a mitochondria-involved mechanism. p53 can bind to Bcl-XL and regulate mitochondrial outer membrane permeabilization status [37]. Therefore, the HMGA2-mediated decrease in the p53 protein levels can repair the mitochondrial membrane damage. A synergetic effect of cell growth was observed by the knockdown of p53 and HMGA2, further suggesting the cooperation of HMGA2 and p53. Furthermore, a direct interaction between HMGA2 and p53 was reported. Whether such an interaction can interfere with the p53 and Bcl-XL complex requires investigation. Additionally, our RNA sequencing result indicates significant changes in RAS signaling and Wnt signaling pathways in HMGA2 overexpressed cells. RAS was identified as one of the multiple downstream targets of Wnt/β-catenin signaling [38]. The activation of Wnt/β-catenin and RAS activity protects mitochondrial function [39]. HMGA2 was identified as a downstream target of RAS signaling [20]. Whether HMGA2 overexpression led to the feedback activation of Wnt/β-catenin and RAS activity to improve mitochondrial function should be further clarified. It was worthy to be noted that p53 mutation was frequently observed and about 50 % frequency in colorectal cancers based on the TP53 alteration data accessed from the IARC TP53 database (http://www-p53.iarc.fr/) [40]. The outcomes of p53 mutants are gain of function or loss of tumor suppressor activity to support tumor progression in various aspects [41]. It was reported that p53 mutants enhance cancer cell proliferation and migration by recruitment with transcription factors to promote oncogenes expression [42]. Notably, p53 mutant can inhibit the expression of let-7, which is an important tumor suppressor gene to suppress tumor growth. While, HMGA2 was a direct target of let-7 in colon cancer cells [43].Thus, it was speculated and reasonable that p53 inhibitors and HMGA2 knockdown have the synergy effects to suppress cancer cell proliferation and migration in our experimental results. On the other hand, mutant p53 can induce chromatin remodeling through binding SWI/SNF chromatin remodeling complex to alter nucleosome occupancy [44,45]. HMGA2 was a chromatin binding factor and can alter chromatin structure through DNA or protein binding. It will be of worthy to clarify in the future whether HMGA2 and mutant p53 form complex to remodel chromatin structure.

Of note is that there have several limitations in our study. The first is that few clinical samples were used. Although the higher expression of HMGA2 was observed in cancer than adjacent tissue, more samples would be necessary to solid the results. The second is that the lack of therapeutic clinical information. The evaluation of overall survival and disease-free survival will be important. Also, the correlation analysis between HMGA2 expression and patients survival would benefit the importance of HMGA2 in colorectal cancer therapy. The third is the insufficiency of detailed molecular mechanism of HMGA2 in cooperation with p53 or mutant p53 and the explore of this point will shed light on targeting investigation for the cancer therapy.

In summary, our results showed that HMGA2 is overexpressed in colorectal cancer and HMGA2 regulates colorectal cancer cell growth. HMGA2 overexpression alleviates cell death by activating the p53 signaling pathway after PTX treatment. Our results highlight the importance of HMGA2 function and can help determine potential therapeutic targets for colorectal cancer treatment.

## Source of funding

This work was supported by Ningbo Top Medical and Health Research Program (No. 2023020612),the project of Ningbo leading Medical & healthy Discipline (2022-S04),Medical Scientific Research Foundation of Zhejiang Province (2022KY1103),Ningbo Natural Science Foundation(2023J153)

## CRediT authorship contribution statement

**Haizhong Jiang:** Writing – original draft, Investigation. **Xueying Li:** Software, Formal analysis, Data curation. **Feng Zhou:** Software, Methodology. **Yang Xi:** Supervision, Methodology. **Guoqiang Xu:** Writing – review & editing, Supervision, Project administration, Conceptualization.

## Declaration of competing interest

The authors declare that they have no known competing financial interests or personal relationships that could have appeared to influence the work reported in this paper.
