# Supplementary figures and images for: HMGA2 promotes resistance against paclitaxel by targeting the p53 signaling pathway in colorectal cancer cells
Source: Heliyon. 2024 May 20;10(11):e31431. doi: 10.1016/j.heliyon.2024.e31431 (PMC11154217; doi:10.1016/j.heliyon.2024.e31431)

Uncropped Western Blot

Fig.1

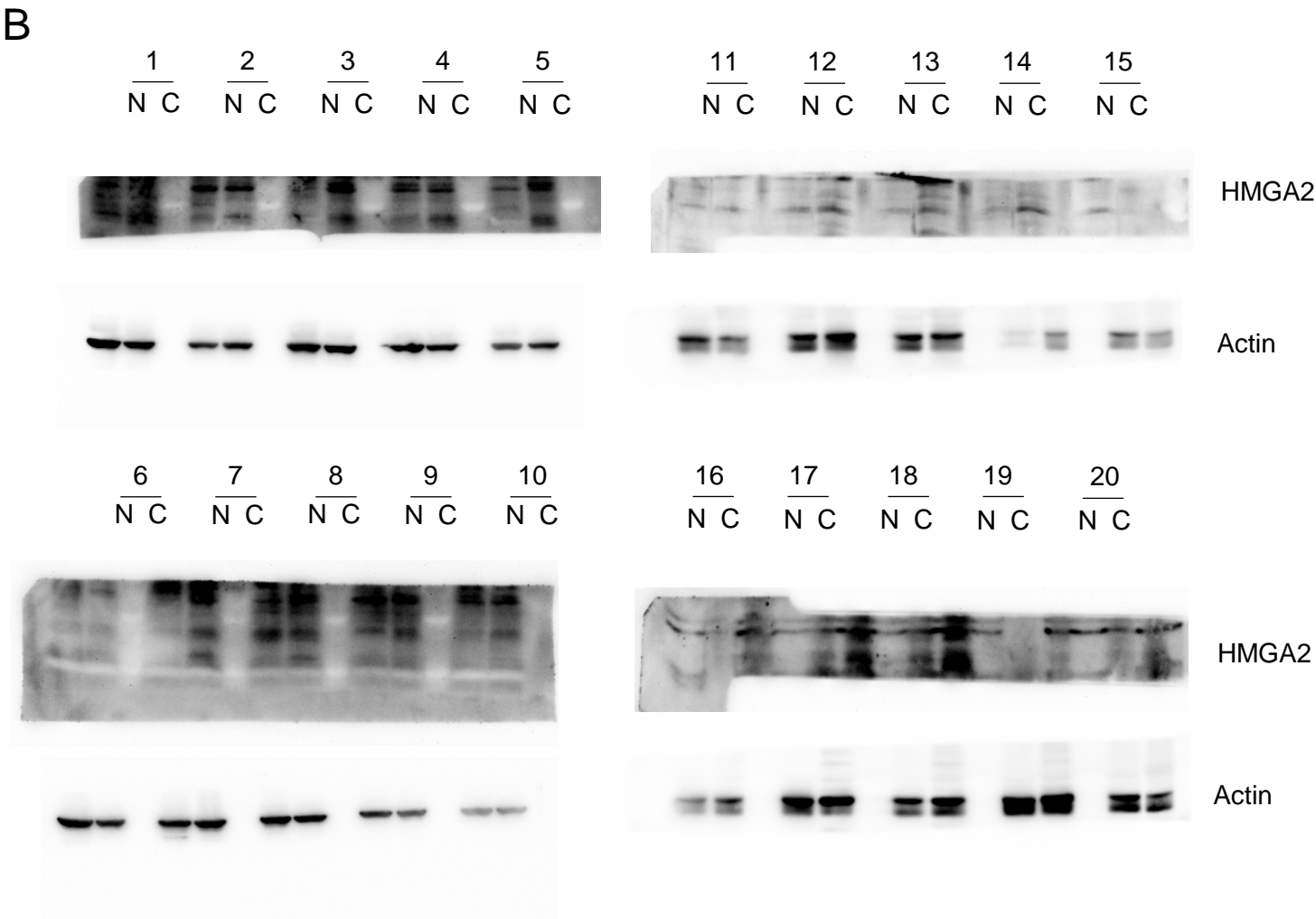

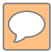

**Fig.1**

**C**

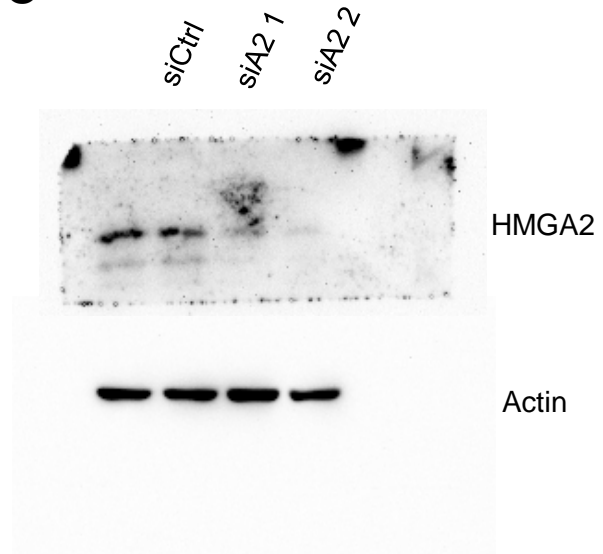

**D**

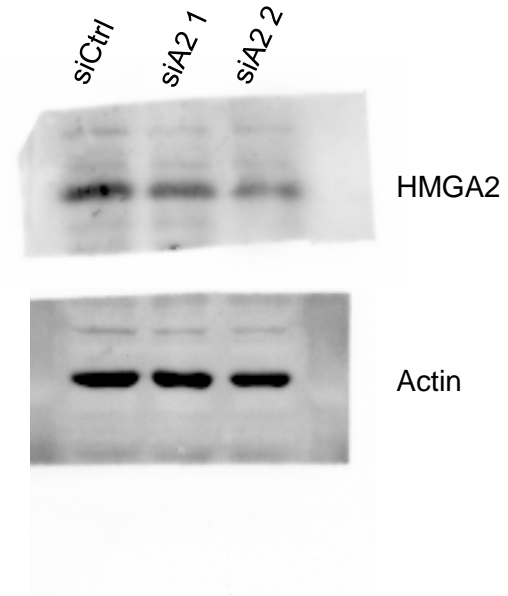

Fig.2

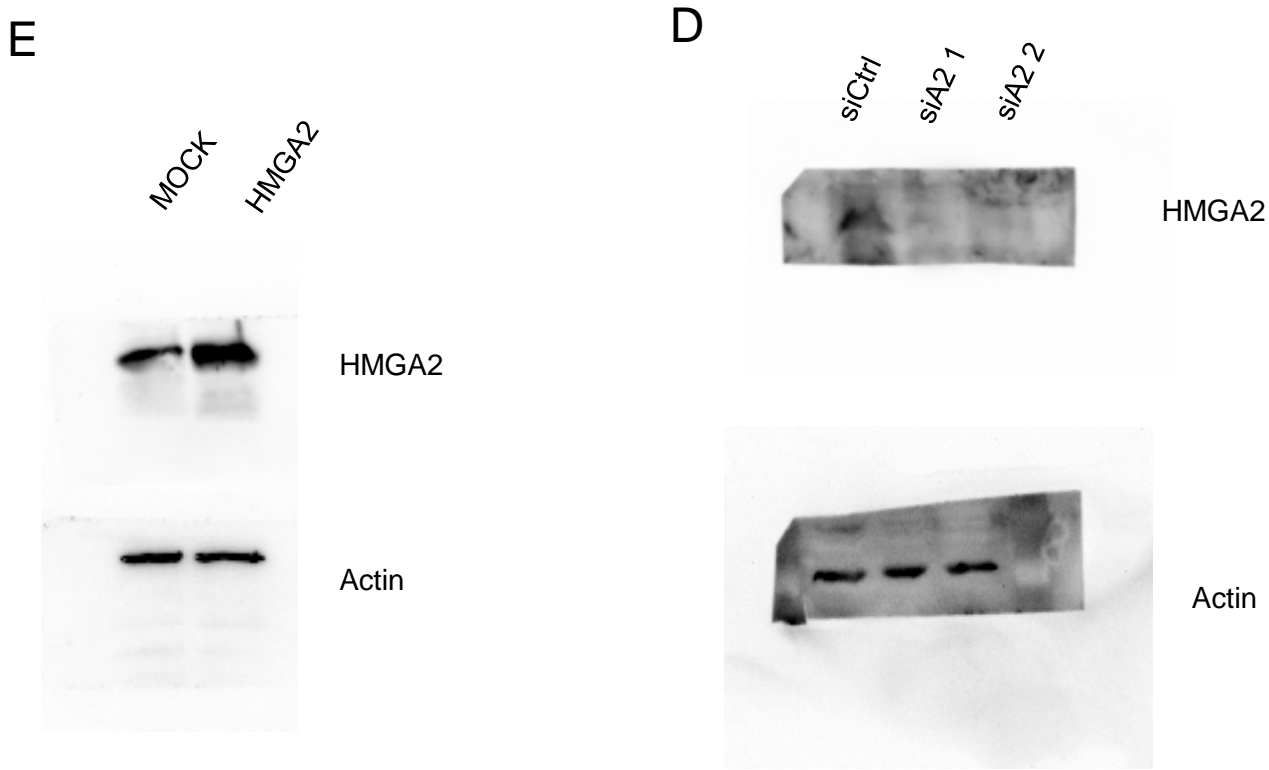

**Fig.4**

**D**

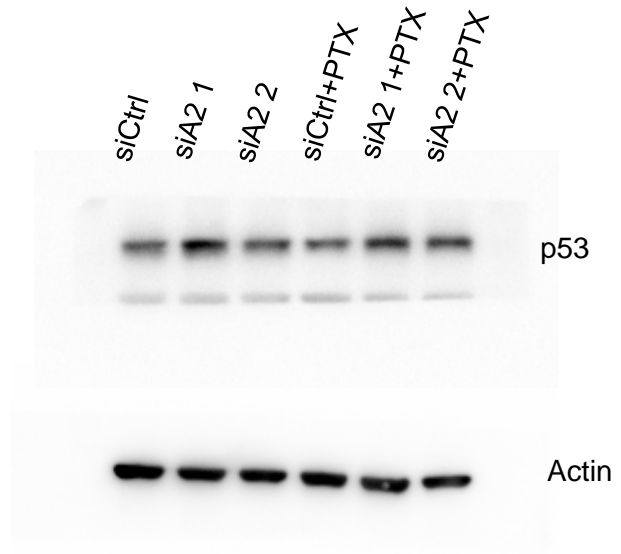

**E**

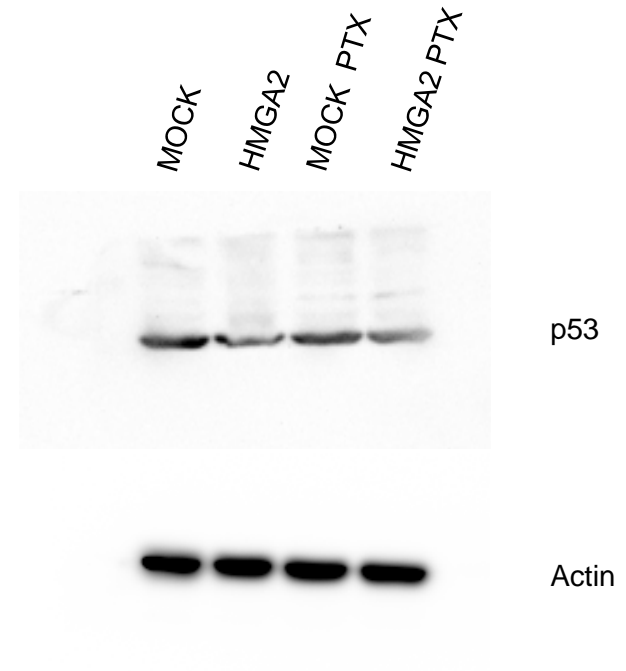

Supplement: Multimedia component 1 [file mmc1.pdf]
